# A new species of the genus *Cerapanorpa* (Mecoptera, Panorpidae) from the eastern Bashan Mountains

**DOI:** 10.3897/BDJ.9.e72451

**Published:** 2021-09-27

**Authors:** Kai Gao, Bao-Zhen Hua

**Affiliations:** 1 Entomological Museum, College of Plant Protection, Northwest A&F University, Yangling 712100, Shaanxi, China Entomological Museum, College of Plant Protection, Northwest A&F University Yangling 712100, Shaanxi China

**Keywords:** alpine zone, biodiversity, China, Panorpidae, scorpionfly

## Abstract

**Background:**

*Cerapanorpa* Gao, Ma & Hua, 2016, an endemic genus of Panorpidae in central China’s mountain regions, currently comprises 21 described species. Recently, the short-horned scorpionfly *C.brevicornis* (Hua & Li, 2007) was confirmed to contain two valid species by phylogeographic and morphological data. Individuals from the highlands of the eastern Bashan Mountains were suggested as a good species, separated from the original short-horned *C.brevicornis*.

**New information:**

*Cerapanorpaalpina***sp. nov.** was described from the alpine zone of the eastern Bashan Mountains in central China. The new species differs from its congeners by the following combination of characters: male bearing an extra-short anal horn on posterior margin of tergum VI; paramere elongate, extending beyond the median tooth of gonostylus and curved laterally at basal half; female medigynium slightly constricted medially without dorsal basal plate. The species number of *Cerapanorpa* is raised to 22.

## Introduction

The single-horned scorpionfly genus *Cerapanorpa* Gao, Ma & Hua, 2016 was erected with *Panorpaobtusa* Cheng, 1949 as the type species (Gao et al. 2016). Soon, the genus was taxonomically revised, based on comparative morphology, molecular phylogeny, cytogenetics and geographical data (Gao and Hua 2019, Miao et al. 2019). *Cerapanorpa* is distinguished from other genera of Panorpidae by male adults having a finger-like anal horn on the posterior margin of tergum VI and female medigynium bearing paired basal plates on both sides of the main plate and a well-developed elongated rod-like axis (Gao and Hua 2019). The anal horn as a clamping structure was used to clamp female’s abdominal segment VIII to maintain copulation (Tong et al. 2018). The monophyly of *Cerapanorpa* was confirmed by morphological (Ma et al. 2012, Wang and Hua 2021) and molecular phylogenetic analyses (Miao et al. 2017, Miao et al. 2019).

The genus *Cerapanorpa* currently consists of 21 described species, which are endemic to the montane archipelagos of central China’s mountains (Gao and Hua 2019). The suitable habitats are universally cool during the imaginal flight period (Gao and Hua 2019, Gao et al. 2020). The islands-like distribution pattern and cool-adapted preference make this group of insects an ideal model to explore the biological response of montane species under climate changes (Gao et al. 2021). The short-horned scorpionfly, *C.brevicornis* (Hua & Li, 2007), is noticeable for an extra-short anal horn on the sixth tergum in males and is widely distributed in the ‘sky islands’ of central China’s mountains (Li et al. 2007, Gao and Hua 2019). Recently, *C.brevicornis* has been confirmed to contain two valid species, based on phylogeographic and morphological evidence (Gao et al. 2021). Individuals from the highlands of the eastern Bashan Mountains (EBMs) were suggested as a good species, separated from the original short-horned *C.brevicornis* (Gao et al. 2021) and, hereby, are described as a new species, *Cerapanorpaalpina* **sp. nov.**, increasing the species number of *Cerapanorpa* to 22.

## Materials and methods

**Figure 1.**
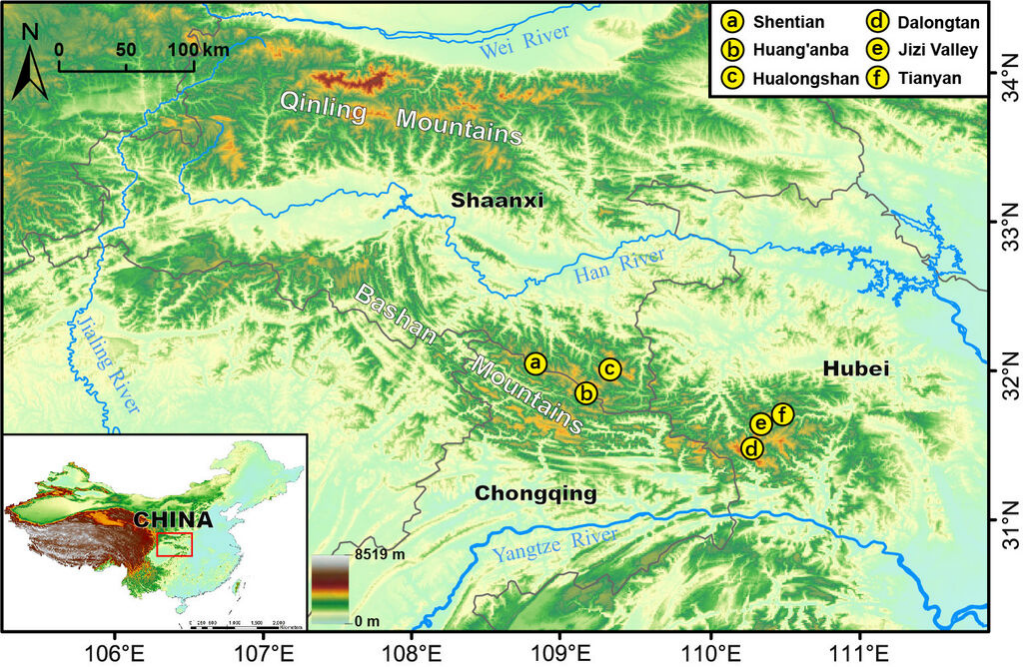
Distribution map of *Cerapanorpaalpina* sp. nov.

Adult scorpionflies were caught with collecting nets from the eastern Bashan Mountains in central China (Fig. 1) and are preserved in 75% or 95% ethanol at the Entomological Museum, Northwest A&F University, China (**NWAU**). Genitalia were dissected under a Nikon SMZ 1500 Stereoscopic Zoom microscope. Male aedeagus and female medigynium were macerated in 5% sodium hydroxide (NaOH) for 3 min and then rinsed with water. Photographs of adult habitus were taken with a Nikon D7100 digital camera and pictures of portions were taken using a scientific digital micrography system ZEISS SteREO Discovery.V20, equipped with an auto-montage imaging system (AxioCam IC). Wings were measured using Imaris v.7.4.2 (Bitplane, Switzerland). The distribution map was generated by ArcGIS v.10.2 (ESRI, Redlands, CA). All pictures were assembled with Adobe Photoshop CS6. Morphological terminology follows Gao et al. (2016), Gao and Hua (2019), and Li et al. (2021).

## Taxon treatments

### Cerapanorpa alpina sp. n.

0F7B3FD7-8F58-5B70-942C-E169170DD893

urn:lsid:zoobank.org:act:14131C38-5657-40F4-8ACE-3AB4FCF9601E

#### Materials

- **Type status:**Holotype. **Occurrence:** recordedBy: Kai Gao, Yuan Hua, Yu-Ru Yang; individualCount: 1; sex: male; lifeStage: adult; **Taxon:** scientificName: *Cerapanorpaalpina*; class: Insecta; order: Mecoptera; family: Panorpidae; genus: Cerapanorpa; **Location:** continent: Asia; country: China; stateProvince: Chongqing; county: Chengkou; locality: Huang'anba; verbatimElevation: 2380 m a.s.l.; verbatimCoordinates: 31°50′43′′N 109°11′23′′E; **Identification:** identifiedBy: Kai Gao; **Event:** year: 2018; month: 6; day: 23; **Record Level:** institutionCode: NWAU; basisOfRecord: PreservedSpecimen
- **Type status:**Paratype. **Occurrence:** recordedBy: Kai Gao, Yuan Hua, Yu-Ru Yang; individualCount: 41; sex: 18 males, 23 females; lifeStage: adult; **Taxon:** scientificName: *Cerapanorpaalpina*; class: Insecta; order: Mecoptera; family: Panorpidae; genus: Cerapanorpa; **Location:** continent: Asia; country: China; stateProvince: Chongqing; county: Chengkou; locality: Huang'anba; verbatimElevation: 2380 m a.s.l.; verbatimCoordinates: 31°50′43′′N 109°11′23′′E; **Identification:** identifiedBy: Kai Gao; **Event:** year: 2018; month: 6; day: 23; **Record Level:** institutionCode: NWAU; basisOfRecord: PreservedSpecimen
- **Type status:**Paratype. **Occurrence:** recordedBy: Kai Gao, Yu-Ru Yang; individualCount: 42; sex: 17 males, 25 females; lifeStage: adult; **Taxon:** scientificName: *Cerapanorpaalpina*; class: Insecta; order: Mecoptera; family: Panorpidae; genus: Cerapanorpa; **Location:** continent: Asia; country: China; stateProvince: Shaanxi; county: Langao; locality: Shentian; verbatimElevation: 2430 m a.s.l.; verbatimCoordinates: 32°02′36′′N 108°49′08′′E; **Identification:** identifiedBy: Kai Gao; **Event:** year: 2018; month: 6; day: 21; **Record Level:** institutionCode: NWAU; basisOfRecord: PreservedSpecimen
- **Type status:**Paratype. **Occurrence:** recordedBy: Kai Gao; individualCount: 25; sex: 12 males, 13 females; lifeStage: adult; **Taxon:** scientificName: *Cerapanorpaalpina*; class: Insecta; order: Mecoptera; family: Panorpidae; genus: Cerapanorpa; **Location:** continent: Asia; country: China; stateProvince: Shaanxi; county: Pingli; locality: Hualongshan; verbatimElevation: 2160 m a.s.l.; verbatimCoordinates: 32°00′23′′N 109°19′25′′E; **Identification:** identifiedBy: Kai Gao; **Event:** year: 2019; month: 7; day: 16; **Record Level:** institutionCode: NWAU; basisOfRecord: PreservedSpecimen
- **Type status:**Paratype. **Occurrence:** recordedBy: Kai Gao, Yu-Ru Yang; individualCount: 23; sex: 9 males, 14 females; lifeStage: adult; **Taxon:** scientificName: *Cerapanorpaalpina*; class: Insecta; order: Mecoptera; family: Panorpidae; genus: Cerapanorpa; **Location:** continent: Asia; country: China; stateProvince: Hubei; county: Shennongjia; locality: Tianyan; verbatimElevation: 2250 m a.s.l.; verbatimCoordinates: 31°42′58′′N 110°21′35′′E; **Identification:** identifiedBy: Kai Gao; **Event:** year: 2018; month: 7; day: 1; **Record Level:** institutionCode: NWAU; basisOfRecord: PreservedSpecimen
- **Type status:**Paratype. **Occurrence:** recordedBy: Kai Gao, Yu-Ru Yang; individualCount: 81; sex: 35 males, 46 females; lifeStage: adult; **Taxon:** scientificName: *Cerapanorpaalpina*; class: Insecta; order: Mecoptera; family: Panorpidae; genus: Cerapanorpa; **Location:** continent: Asia; country: China; stateProvince: Hubei; county: Shennongjia; locality: Dalongtan; verbatimElevation: 2180 m a.s.l.; verbatimCoordinates: 31°29′39′′N 110°18′10′′E; **Identification:** identifiedBy: Kai Gao; **Event:** year: 2018; month: 7; day: 3; **Record Level:** institutionCode: NWAU; basisOfRecord: PreservedSpecimen
- **Type status:**Paratype. **Occurrence:** recordedBy: Ji-Shen Wang, Yuan Hua; individualCount: 6; sex: 4 males, 2 females; lifeStage: adult; **Taxon:** scientificName: *Cerapanorpaalpina*; class: Insecta; order: Mecoptera; family: Panorpidae; genus: Cerapanorpa; **Location:** continent: Asia; country: China; stateProvince: Hubei; county: Shennongjia; locality: Jizi Valley; verbatimElevation: 1800 m a.s.l.; verbatimCoordinates: 31°32′06′′N 110°19′59′′E; **Identification:** identifiedBy: Kai Gao; **Event:** year: 2016; month: 6; day: 6; **Record Level:** institutionCode: NWAU; basisOfRecord: PreservedSpecimen

#### Description

**Figure 2.**
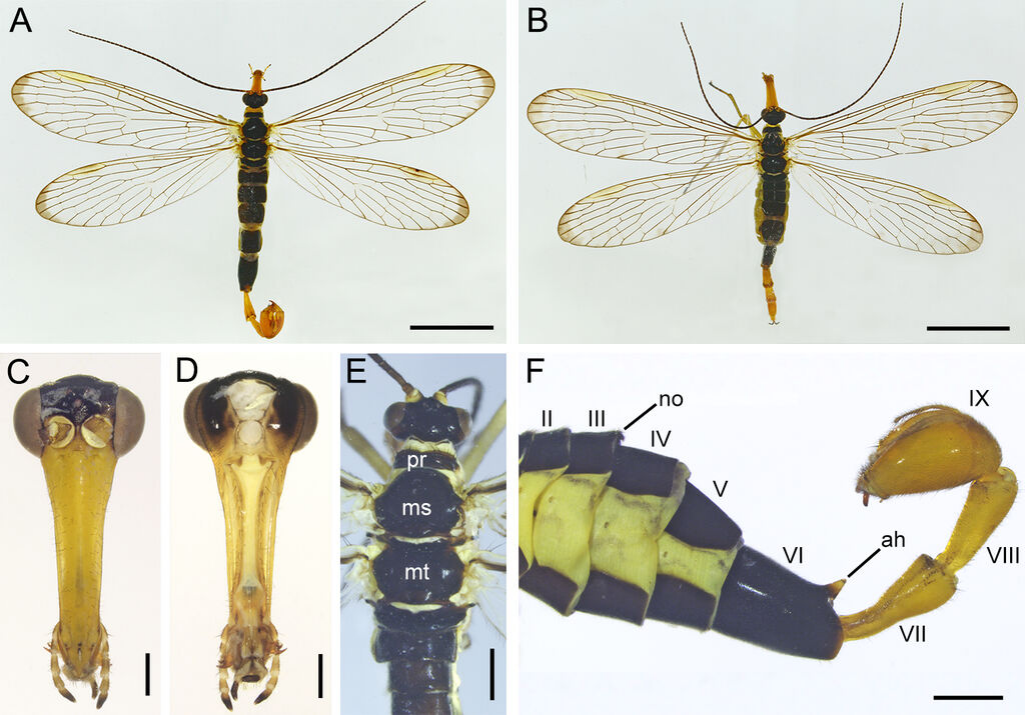
Adults of *Cerapanorpaalpina* sp. nov. **A, B.** Male and female habitus in dorsal views; **C, D.** male head in frontal and posterior views; **E.** male dorsum of head and thorax; **F.** male abdomen in lateral view. Abbreviations: ah, anal horn; ms, mesonotum; mt, metanotum; no, notal organ; pr, pronotum. Abdominal segments are indicated by Roman numerals. Scale bars: A, B = 5 mm; C, D = 0.5 mm; E, F = 1 mm.

**Male**: Frons, vertex, occiput and postgena brownish-black (Fig. 2C and D). Rostrum uniformly yellowish-brown, mandibles brown. Antennae black with 38–42 flagellomeres. Pro-, meso- and metanotum black, bearing black stout setae anteriorly (Fig. 2E). Pleura and legs yellowish-brown. Forewing length 13.87–15.49 mm, width 3.34–3.62 mm. Wing broad and membrane hyaline; apical band only with dark grey trace at apical region (Figs. 2A and 4A). Hindwing length 12.30–13.85 mm, width 3.10–3.45 mm, similar to forewing in shape and venation. Terga I–V entirely black. Notal organ on the posterior margin of tergum III semicircular, not prominent; postnotal organ on tergum IV small and hook-shaped. Tergum VI with a very short finger-like anal horn on posterior margin (Fig. 2F). Segments VII and VIII elongate, constricted basally and thicker gradually toward apex.

**Figure 3.**
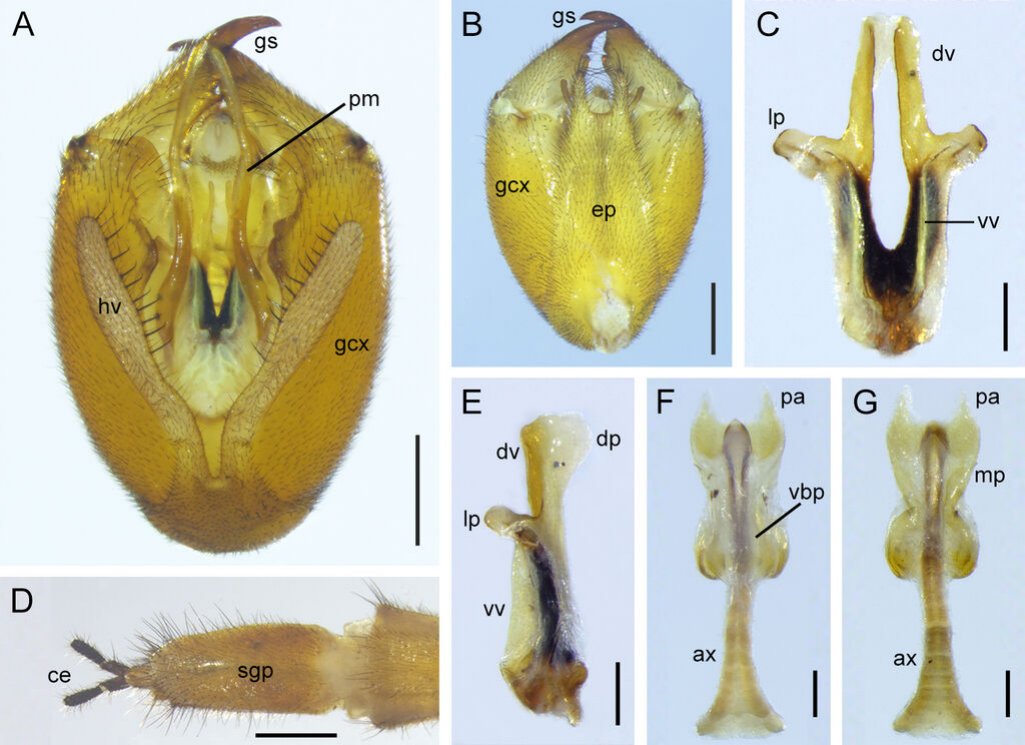
Genitalia of *Cerapanorpaalpina* sp. nov. **A, B.** Male genital bulb in ventral and dorsal views; **C, E.** aedeagus in ventral and lateral views; **D.** female terminalia in ventral view; **F, G.** medigynium in ventral and dorsal views. Abbreviations: ax, axis; ce, cercus; dp, dorsal process; dv, dorsal valve; ep, epandrium; gcx, gonocoxite; gs, gonostylus; hv, hypovalve; lp, lateral process; mp, main plate; pa, posterior arm; pm, paramere; sgp, subgenital plate; vbp, ventral basal plate; vv, ventral valve. Scale bars: A, B, D = 0.5 mm; C, E–G = 0.2 mm.

Male genitalia: Genital bulb elliptical, yellowish-brown (Fig. 3A). Epandrium (tergum IX) broad at base, gradually narrowing towards apex, with deep U-shaped emargination terminally (Fig. 3B). Hypovalve slender, bearing several short stout setae along inner margin (Fig. 3A). Gonocoxite with two subtriangular protuberances on ventral submedian margin (Fig. 3A). Gonostylus bearing indistinct median tooth and prominent basal process on inner margin. Paramere elongate, extending beyond median tooth of gonostylus, curved laterally at basal half and bearing numerous dense spines along inner margin (Fig. 3A). Aedeagus almost straight; dorsal valves short and inflated apically; ventral valves elongate and membranous; lateral process prominent and curved ventrally (Fig. 3C and E).

**Figure 4.**
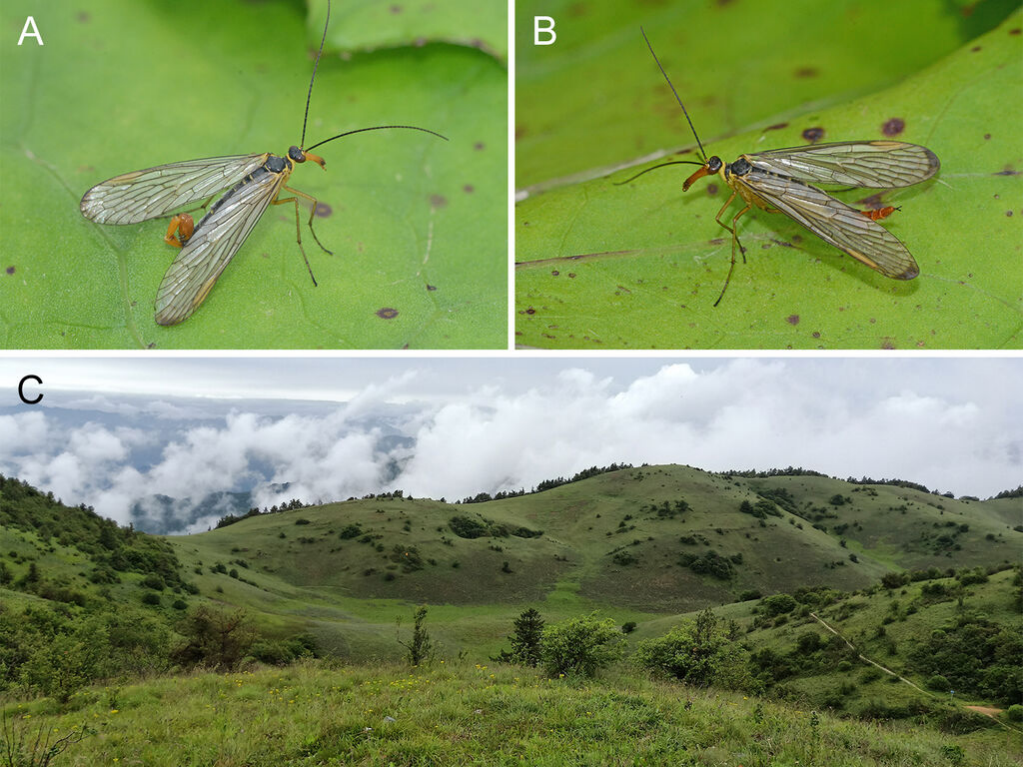
Adult habitus and habitat of *Cerapanorpaalpina* sp. nov. **A.** Male; **B.** female; **C.** habitat in the Shentian Alpine Meadow, Langao County, Shaanxi, China.

**Female**: Head, thorax and abdomen similar to those of males in colouration and pattern (Figs. 2B and 4B). Head length 3.85–4.26 mm. Forewing length 14.59–16.22 mm, width 3.54–3.96 mm. Hindwing length 13.28–14.85 mm, width 3.10–3.45 mm.

Female genitalia: Subgenital plate nearly trapezoidal, shallowly emarginate terminally, with long bristles on lateral distal part (Fig. 3D). Medigynium with main plate twice as long as wide and slightly constricted medially (Fig. 3G). Paired posterior arms forming subquadrate emargination (Fig. 3F and G). Ventral basal plate translucent, covering approximately two-thirds of the main plate; dorsal basal plate lacking (Fig. 3G). Axis bifurcate, elongate, extending anteriorly over half its length beyond main plate (Fig. 3F and G).

#### Diagnosis

The new species can be distinguished from its congeners by the following combination of characters: postgena brownish-black; male tergum VI with an extra-short anal horn on posterior margin; paramere exceeding beyond the median tooth of gonostylus and curved laterally in basal half; female subgenital plate nearly trapezoidal; medigynium lacking dorsal basal plate.

#### Etymology

The specific epithet ‘*alpina*’ is derived from the Latin adjective ‘*alpinus*’, referring to the high elevation habitats in the eastern Bashan Mountains.

#### Distribution

China (Chongqing, Shaanxi and Hubei provinces).

#### Habitat

The new species was found exclusively in the eastern Bashan Mountains with an altitude of above 1800 m and was especially rich on the alpine shrub meadows of mountaintops (Fig. 4C).

#### Remarks

The new species is closely allied to *C.brevicornis* (Hua & Li, 2007), but differs from the latter by the following characters: postgena brownish-black (cf. yellowish-brown); male paramere elongate, exceeding beyond the median tooth of gonostylus and curved laterally in basal half (cf. shorter and not curved in basal half); female subgenital plate nearly trapezoidal (cf. elliptical); main plate of female medigynium slightly constricted medially (cf. narrower at base and gradually broadened towards the apex).

## Discussion

*Cerapanorpaalpina* sp. nov. and *C.brevicornis* (Hua & Li, 2007) share a most recent common ancestor (Gao et al. 2021). The two sibling species are very similar in gross appearance, both possessing an extra-short anal horn on the posterior margin tergum VI in males. However, *C.alpina* can be separated from *C.brevicornis* in the shape and size of genital structures (e.g. hypovalve, paramere, aedeagus, subgenital plate and medigynium). The mean genetic distance of the *COI* gene between the two species is 0.043 (Gao et al. 2021), much higher than the criterion of 2% (maximum intraspecific divergence) (Hebert et al. 2003), reinforcing the status of the new species. Furthermore, the two short-horned scorpionfly species have independent evolutionary trajectories and separately restricted in different interglacial refugia for a relatively long time (Gao et al. 2021). Finally, *C.brevicornis* and *C.alpina* also have different distributions, the former is widely distributed in the Qinling, Bashan and Minshan mountains, whereas the latter is only found in the eastern Bashan Mountains.

*Cerapanorpaalpina* sp. nov. is endemic to the alpine zone of the EBMs, an important climate refugium and a centre of endemism for montane species (Gao et al. 2021). Recently, many new endemic species of Panorpidae were reported from this region, including *Panorpabiclada* Zhang & Hua, 2012, *P.bashanicola* Hua, Tao & Hua, 2018, *P.gaokaii* Li, Wang & Hua, 2021, *P.huayuani* Li, Wang & Hua, 2021, *Sinopanorpadigitiformis* Huang & Hua, 2008, *S.nangongshana* Cai & Hua, 2008, *Dicerapanorpashennongensis* Zhong & Hua, 2013, *D.hualongshana* Hu & Hua, 2019, *Cerapanorpaprotrudens* Gao, Ma & Hua, 2016 and *Megapanorpagaokaii* Wang & Hua, 2019. Scorpionflies inhabiting the ‘sky islands’ of the EBMs (e.g. alpine shrub meadows) generally have relatively restricted distribution and cool-climate preference. These fragmented highlands may provide suitable microhabitats for montane species to survive past climate fluctuations (Gao et al. 2021). This could be a major reason why the EBMs possess extraordinarily high biodiversity and endemicity. In this sense, the alpine zone of the EBMs should be considered as a high-priority region for montane biodiversity conservation.

## Supplementary Material

XML Treatment for Cerapanorpa alpina
